# Complete Genome Sequences of Four Mycobacteriophages Involved in Directed Evolution against Undisputed *Mycobacterium abscessus* Clinical Strains

**DOI:** 10.3390/microorganisms12020374

**Published:** 2024-02-11

**Authors:** Juan Carlos Cao Yao, Damir Garcia Cehic, Josep Quer, Jesús Navas Méndez, Alexis Dorta Gorrín, Lorena García Hevia, María Teresa Tórtola Fernández

**Affiliations:** 1 Department of Molecular Biology and Biomedicine, University of Cantabria, 39011 Santander, Spainalexis.dorta@alumnos.unican.es (A.D.G.); lgarcia@idival.org (L.G.H.); 2 Liver Diseases-Viral Hepatitis, Liver Unit, Vall d’Hebron Institut de Recerca (VHIR), Vall d’Hebron Barcelona Hospital Campus, Hospital Universitari Vall d’Hebron, Passeig Vall d’Hebron 119-129, 08035 Barcelona, Spain; damir.garcia@vhir.org (D.G.C.); josep.quer@vhir.org (J.Q.); 3 CIBER de Enfermedades Hepáticas y Digestivas, Instituto de Salud Carlos III, 28029 Madrid, Spain; 4 Biochemistry and Molecular Biology Department, Universitat Autònoma de Barcelona, 08193 Bellaterra, Spain; 5 Instituto de Investigación Valdecilla (IDIVAL), 39011 Santander, Spain; 6 Mycobacteria Unit, Clinical Laboratories, Microbiology Service, Hospital Universitari Vall d’Hebron, Universitat Autònoma de Barcelona, 08035 Barcelona, Spain

**Keywords:** *Mycobacterium*, temperate, lytic, cluster, gene, function

## Abstract

Phage therapy is still in its infancy, but it is increasingly promising as a future alternative for treating antibiotic-resistant bacteria. To investigate the effect of phages on *Mycobacterium abscessus* complex (MABC), we isolated 113 environmental phages, grown them to high titres, and assayed them on MABC clinical strains through the spot test. Of all the phages, only 16 showed killing activity. Their activity was so temperate to MABC that they could not generate any plaque-forming units (PFUs). The Appelmans method of directed evolution was carried out to evolve these 16 phages into more lytic ones. After only 11 of 30 rounds of evolution, every single clinical strain in our collection, including those that were unsusceptible up to this point, could be lysed by at least one phage. The evolved phages were able to form PFUs on the clinical strains tested. Still, they are temperate at best and require further training. The genomes of one random parental phage and three random evolved phages from Round 13 were sequenced, revealing a diversity of clusters and genes of a variety of evolutionary origins, mostly of unknown function. These complete annotated genomes will be key for future molecular characterisations.

## 1. Introduction

A wide variety of microorganisms infiltrate the airways of cystic fibrosis (CF) patients [1,2,3]. Amongst them, the prevalence of *Mycobacterium abscessus* complex (MABC) is steadily increasing. This complex is a subset of nontuberculous mycobacteria (NTM) consisting of three different subspecies: *abscessus*, *massiliense*, and *bolletii*. Once an environmental saprophyte, MABC has evolved into an opportunistic pathogen, mainly targeting immunocompromised individuals [4].

Since its discovery a century ago, phagotherapy has primarily been applied in Eastern Europe under compassionate use. However, as bacteria become increasingly resistant to drugs, hospitals around the world are now opening their doors to this alternative treatment on a compassionate basis [5]. 

From 2000 to 2019, compassionate phagotherapy has been given to almost 2000 patients to treat a wide range of infections. As of April 2019, out of 29 published cases of infections including *A. baumannii*, *K. pneumoniae*, *S. aureus*, *P. aeruginosa*, *E. coli*, *Proteus*, *Streptococcus*, *A. xylosoxidans*, *Enterococcus*, and *Enterobacter*, phage therapy achieved a success rate of nearly 76% [6]. A summary of phage treatment cases at the Hospital of Villeneuve Saint Georges, France, from 2006 to 2018, against *S. aureus*, *E. coli*, *P. aeruginosa*, and *Proteus*, showed success in 14 of 15 cases [7]. 

The Actinobacteriophage Database has so far registered 13,238 mycobacteriophages, of which 2295 phages are sequenced. Moreover, 13,191 of the mycobacteriophages (99.6%) have been isolated using *Mycobacterium smegmatis* mc^2^ 155 as host. Only four mycobacteriophages have been isolated using MABC as host, one phage (Maco6) infecting *M. abscessus* C107, two phages (Isca and Halo (also known as Diddy or Galen)) infecting *M. abscessus* GD01, and one phage (Adler) infecting *M. abscessus* subsp. *bolletii* F1660. Phage Elmo, isolated from *Mycobacterium smegmatis* mc^2^ 155, also infects *M. abscessus* GD02 [8].

In 2018, long-term antibiotic therapy made no difference in a 30-year-old patient’s fracture-related infections due to extremely drug-resistant strains of *K. pneumoniae*. Bacterial isolates were sent to the George Eliava Institute of Bacteriophage, Microbiology, and Virology. Screening identified phage vB_KpnM_M1 (M1) as highly active. M1 had been collected from sewage waters in Tbilisi a decade earlier. M1 was active against a wide range of *Klebsiella* species. However, to prevent bacterial phage resistance, the Appelmans method of directed evolution was carried out. The resulting preadapted phage had a Thr281Arg missense mutation in the loop region of the hinge connector of the distal tail fibre protein, which may explain its higher efficacy in lysing the patient’s strains. By Belgian law on phage therapy, there cannot be genes encoding for toxic, lysogenic, or antibiotic resistance-related proteins, which phage M1 did not have. Following a bone graft surgery, preadapted M1 phages were locally administered into the infected femur directly through a catheter, in combination with meropenem and colistin. A course of ceftazidime/avibactam later followed, accompanied by tigecycline and ciprofloxacin. Three months later, the patient was virtually healed, with restored clinical, biological, microbiological, and radiological conditions [9]. 

In 2019, a success story was reported of a 15-year-old CF patient who had a double-lung transplant after eight years of unsuccessful antibiotic regimen against *Pseudomonas aeruginosa* and *M. abscessus* subsp. *massiliense*. After transplant, antibiotic treatment was resumed, but MABC still disseminated. High-throughput screening of over 10,000 *M. smegmatis*-isolated phages led to the discovery of phages Muddy, which infects GD01 effectively, ZoeJ, which infects GD01 less well, and BPs. BPs was engineered to its lytic derivative BPsΔ33HTH. Both BPs and its lytic derivative hardly infect GD01. However, ZoeJ had its repressor gene 45 accurately deleted through Bacteriophage Recombineering of Electroporated DNA (BRED) to create its lytic derivative ZoeJΔ45, and host range mutant BPs33ΔHTH-HRM10 was found, both of which effectively kill GD01. Muddy was found in Durban, South Africa. ZoeJ was found in Providence, RI, USA. BPs was found in Pittsburgh, PA, USA. The cocktail of phages Muddy, ZoeJΔ45, and BPs33ΔHTH-HRM10 was compassionately administered intravenously alongside an unbroken course of antibiotic treatment. The patient was soon healed [8,10]. The single bacteriophage Muddy was employed in June 2021, accompanied by a course of antibiotics, to treat a case of disseminated *M. chelonae* infection, with outstanding clinical outcomes [11]. These examples stand in stark contrast to the case of an 81-year-old patient with *M. abscessus* lung disease who was treated with the same three-phage cocktail but with utter failure. Multi-antibiotic therapy remained the only choice [12]. 

A study found that the Center for Innovative Phage Applications and Therapeutics (IPATH), USA, received 644 requests for phage treatment for bacterial infections from 1 June 2018 to 30 April 2020, of which 90 requests (14%) were for treatment against mycobacteria. *P. aeruginosa* (14.3%), *S. aureus* (12%), and *M. abscessus* (7.3%) made up the top three pathogens requested. Out of the 47 requests for *M. abscessus*, 18 phages were hunted, nine lytic phages were found, four patients received phage treatment, and three patients were pending phage therapy. More details were not given [7]. 

A 26-year-old male CF patient infected with *M. abscessus*, multidrug-resistant *P. aeruginosa*, and methicillin-resistant *S. aureus* was given multi-antibiotic therapy for almost five years, during which persistent *M. abscessus* subsp. *abscessus* relentlessly exacerbated his lung condition and made a lung transplant impossible. It was found that the patient’s *M. abscessus* clinical isolate could be killed by two host range mutants of the lytic derivative BPsΔ33HTH, BPsD33HTH_HRM10 and BPsD33HTH_HRM^GD03^. D29 is a lytic phage isolated on the host *M. smegmatis* from the UCLA campus in California, USA [8]. Its host range mutant D29_HRM^GD40^ obtained from *M. abscessus* strain GD40 could lyse a number of *M. abscessus* clinical strains. The patient’s strain was highly susceptible to both BPsD33HTH_HRM10 and D29_HRM^GD40^, whether individually or together. A cocktail made of these two phages was finally intravenously administered in September 2020. Over the course of the following year, the *M. abscessus* infection was gradually quelled and the patient soon received a successful lung transplant. Antibiotic treatment was never interrupted [13]. 

A cohort study of an international sample of patients suffering from mycobacterial infections shows a decent phagotherapy success rate of 11 of 20 patients. Five patients had ‘Complex, inconclusive, or incomplete responses’, and four patients had ‘No evident clinical improvement’. All but three of the patients had MABC infections. The phages were selected or engineered to suit the phage sensitivity profiles of individual patient isolates [14].

Since the great Belgian doctor René Appelmans introduced the Appelmans protocol of directed evolution in 1921 [15], directed evolution has been revolutionising a multitude of biotechnological areas, including the engineering of nucleic acids, enzymes, metabolic pathways, genetic circuits, viruses, and whole cells, and is being widely applied in the energy, chemical, pharmaceutical, agriculture, and food industries. However, the greatest contribution of directed evolution is arguably bringing evolutionary theory to real-life practice, proving yet again how theory nourishes experiment. In its essence, directed evolution is an accelerated in vitro simulation of Darwinian evolution, and its underlying principles remain: (1) Creation of an as diverse as possible library of variants by way of mutagenesis or gene recombination; (2) Screening to select the variants closest to the desired characteristics; (3) Iteration of the cycle until the optimal variant is attained. Yet, it is important to keep in mind that directed evolution is not natural selection. Variants created in the laboratory may not be compatible with nature but have industrial uses, and variants that would never arise by random mutagenesis due to molecular obstructions can be forcefully conceived through saturation mutagenesis [16,17].

Viruses replicate fast and thus have a short time frame to adapt to new hosts. It has been empirically observed that 30–50% of random mutations are highly deleterious, 50–70% are virtually inoffensive, and only 0.5–0.01% are advantageous for enzymes in terms of activity or stability gain [18]. Spontaneous mutation rates differ greatly amongst viruses. RNA viruses have a higher rate than DNA viruses, single-stranded viruses have a higher rate than double-stranded viruses, and genome size and mutation rate seem to be negatively correlated [19].

Should viruses rely solely on mutations, evolution would be rather slow indeed. On the contrary, bacteria play a crucial role in viral genome evolution. The exact mechanisms of it are not known, but a few aspects are clear. A cocktail of phages adapts to a refractory bacterial strain principally by spontaneous mutation and recombination [15,20]. Phage genomes have a mosaic configuration due to horizontal genetic transfer that has been occurring ever since the dawn of life. Mosaicism can potentially arise by three mechanisms—homologous recombination, illegitimate recombination, and site-specific recombination—though evidence indicates illegitimate recombination is virtually the sole driver of the pervasive mosaicism observed [21]. A bacterial strain sensitive to several phages in a cocktail provides a unique platform for recombinations that dramatically enrich and potentiate the cocktail in unimaginable ways, way more than mutations alone.

We had previously conducted a thorough investigation of the antibiotic susceptibility profiles of 43 highly refractory MABC clinical strains [22]. In the quest to kill them efficiently, our present research sets out to develop phage therapy. We hunted a rich collection of environmental phages; assayed their activity against the 43 MABC clinical isolates and strain HUMV 29, provided by the Spanish Society of Infectious Diseases and Clinical Microbiology (SEIMC); artificially created a cocktail of phages with enhanced potency over the environmental ones; assayed their new activity against the 44 MABC strains; and characterised a selection of phages through whole-genome sequencing. 

## 2. Materials and Methods

### 2.1. Phage Collection

Over 100 wastewater samples from Hospital Universitario Marqués de Valdecilla (HUMV) and from sewage water samples from the sewage facility of the city of Santander, Spain, and a sewage facility of the city of Valencia, Spain, were collected. Moreover, 10 mL of raw samples were filtered through a 0.45-μm filter and a 0.22-μm filter. One ml of the resulting filtrate was added to 200 μL of *M. smegmatis* mc^2^ 155 suspension in LB broth, mixed with three ml of top agar, overlaid on LB agar and incubated for 48 h at 37 °C. Samples that showed plaque-forming units (PFUs) were further confirmed by the phage activity assay.

### 2.2. Phage Activity Assay

A PFU from the phage isolation plate was picked and suspended in 100 μL LB broth. Moreover, 5 μL of the suspension was transferred and serially diluted in 495 μL LB broth to dilutions 10^0^, 10^−2^, 10^−4^, and 10^−6^. 10 μL of each dilution were added to 200 μL *M. smegmatis* wild-type strain mc^2^ 155 suspension (OD = 0.4), and three ml of top agar were added to the mixture, which was plated on LB agar and incubated for 48 h at 37 °C. If the number of PFUs across the plates was observed to follow the dilution trend, that sample was taken as positive for the presence of the phage and was then amplified.

### 2.3. Phage Amplification

If morphologically distinct PFUs were observed in the phage activity assay, each one was processed as a distinct phage sample. A well-defined PFU was picked and suspended in 10 mL of LB broth and seeded with 1 mL of *M. smegmatis* mc^2^ 155 suspension. The culture was incubated aerobically on an orbital shaker for 24 h at 37 °C. Moreover, 1 mL of *M. smegmatis* mc^2^ 155 suspension was added to the culture, and it was incubated again aerobically on an orbital shaker for 24 h at 37 °C. The process can be repeated one further round to ensure high-titre phage lysates. Finally, the culture was centrifuged at 10,000 rpm and filtered through 0.22-μm filter to yield a pure high-titre phage lysate for storage at –20 or –80 °C.

### 2.4. Phage Titration

From pure amplified phage sample, 10 μL was transferred to 990 μL LB broth to make serial dilutions of 10^0^, 10^−2^, 10^−4^, and 10^−6^. Moreover, 10 μL of each dilution were mixed with 200 μL of *M. smegmatis* mc^2^ 155 suspension and three ml top agar and overlaid on LB agar. The plates were incubated at 37 °C for 48 h. If a trend of PFUs that followed the dilution trend was observed, that sample was considered successfully amplified and titrated. A plate with lacey appearance was chosen to calculate the phage titre.

### 2.5. Spot Test

200 μL of MABC liquid culture (OD = 0.4) were mixed with three ml top agar and poured onto 7H10 or LB agar since it has been observed that the two media favour different morphotypes [23], which may affect phage susceptibility. The plate was incubated at room temperature for 15 min to allow the culture to solidify. 3 μL of each phage were spotted on its designated site in the plate. The plate was incubated at 37 °C for 72 h. If lytic areas—whether cloudy or clear—were observed, the phages were considered active against MABC. 

### 2.6. Activity Assay of MABC-Active Phages on MABC

10 μL of phage that proved active against MABC were mixed with 200 μL of clinical strain HUMV 5, HUMV 20, and HUVH 8862 suspension and three ml top agar and overlaid on LB agar. The plates were incubated at 37 °C for 48 h. If PFUs were observed, titrations on the same clinical strains were performed. 

### 2.7. Directed Evolution

Using environmental parent phages F2, F6, F10, F15, F23, F27, 25/9, 26/9(2), 12/10, 24/10, 14/11, 18/12, 21/12, 29/12, and 9/1, selected for being the only ones showing activity in the spot tests, the Appelmans method of directed evolution was carried out similarly to as described previously [15]. To optimise time and efficiency, six representative MABC clinical strains were selected and divided into two groups (Table 1). Group 1 contained clinical isolates HUMV 5, HUMV 20, and HUVH 8862. Group 2 contained HUMV 25, HUMV 29, and HUVH 7898. Directed evolution was performed for both groups of bacterial strains in parallel but on alternating days.

### 2.8. Isolation of Evolved Phages

Due to time constraints, progeny phages from the latest cocktails, AP13 and AP11′, had to be isolated using serial dilutions as described in Section 2.4., on the faster-growing *M. smegmatis* host rather than on their slower-growing MABC host. Phages were harvested using a toothpick from PFUs that were as morphologically distinct as possible, suspended in 10 mL LB, vortexed, and filtered through a 0.22-μm filter.

### 2.9. DNA Extraction

One parent phage (25/9) and one Group 1 Appelmans Round 13 (AP13) phage (Φ13.3) were randomly chosen for whole-genome sequencing. DNA was extracted using the AllPrep DNA/RNA Mini kit (50) (QIAGEN, Venlo, The Netherlands) and quantitated using the Qubit 3 fluorometer (Invitrogen, Carlsbad, CA, USA). DNA library was prepared using Illumina^®^ DNA Prep, (M) Tagmentation (24 Samples) and Nextera™ DNA CD Indexes (96 Indexes, 96 Samples) (Illumina, San Diego, CA, USA). DNA quality was controlled with TapeStation D1000 Screentape, TapeStation D1000 Sample Buffer, and the TapeStation system (Agilent Technologies, Santa Clara, CA, USA). Genome sequencing was performed with MiSeq Reagent Kit v2 (300-cycles), PhiX Control v3, and MiSeq™ System (2 × 150 bp paired-end reads) (Illumina, San Diego, CA, USA).

### 2.10. Genome Sequencing

Raw reads were quality-controlled using the programme FastQC (Galaxy Version 0.73 + galaxy0), trimmed using the programme Fastp (Galaxy Version 0.23.2 + galaxy0), assembled de novo in GS De Novo Assembler, and evaluated and finished in Consed. Weak regions were resolved with AceUtil. The genomes were aligned against the NCBI database using the BLAST algorithm [24] and against the Actinobacteriophage Database using the BLAST algorithm [25] to determine their cluster.

### 2.11. Functional Annotation

Features to of all the genomes were added on the Phage Evidence Collection and Annotation Network (PECAAN) [26]. Genomes were first auto-annotated using Glimmer [27] and GeneMark [28] and then manually refined and functionally annotated based on the top hits in multiple sequence alignments using NCBI BLASTP [24], PhagesDB BLASTP [25], HHPred [29], and Phamerator [30]. Conserved Domain Database (CDD) [31] hits were used to support HHPred hits further. Aragorn [32] and tRNAscan-SE [33] were applied to scan for the presence of tRNAs and tmRNAs. Genomes were finally deposited in GenBank. 

## 3. Results

### 3.1. Phage Isolation

A total of 113 environmental phage samples were isolated from the host *M. smegmatis* wild-type strain mc^2^ 155, and their titres are presented in Tables S1 and S2. Due to time constraints, directed evolution had to be aborted prematurely, resulting in the isolation of 21 evolved phages in total: Φ11′.1, Φ11′.2, Φ11′.3, Φ11′.4, Φ11′.5, Φ11′.6, Φ11′.7, Φ11′.8, Φ11′.9, Φ11′.10, and Φ11′.11 being isolated from Round 11 of Group 2 (AP11′), and Φ13.1, Φ13.2, Φ13.3, Φ13.4, Φ13.5, Φ13.6, Φ13.7, Φ13.8, Φ13.9, and Φ13.10 being isolated from Round 13 of Group 1. Their titres are yet unknown. 

### 3.2. Spot Test

Spot tests of the phages from Valencia against HUVH MABC strains grown on 7H10 demonstrated that F6, F23, and F27 were the active ones. Clinical strains HUVH 6970, 8130, 8153, 8396, 8874, 8727, 9161, and 9380 proved to be susceptible to at least cocktail AP11. Furthermore, clinical strain HUVH 8261, which remained inactive against all environmental phages tested, finally showed activity to cocktail AP11 (Table S3). 

Spot tests of the phages from Valencia against HUMV MABC strains grown on 7H10 demonstrated that F2, F6, F10, F23, and F27 were the active ones (Table S4). 

Spot tests of the phages from Valencia against HUMV MABC strains grown on LB agar demonstrated that F2, F6, F10, F15, F21, F23, and F27 were the active ones (Table S5). Strain HUMV 1 was found susceptible to at least cocktail AP11.

Spot tests of the phages from Santander against HUMV MABC strains grown on LB agar demonstrated that 25/9, 26/9(2), 12/10, 24/10, 14/11, 18/12, 21/12, 29/12, and 9/1 were active (Table S6). Strain HUVH 8862, which was resistant to all Valencia phages, was sensitive to these Santander phages. 

### 3.3. Activity Assay of MABC-Active Phages on MABC

None of the environmental phages were lytic enough to produce any PFUs against MABC (i.e., titre = 0 PFU/mL), and thus, the efficiency of plating (EOP) is 0. They could only form lytic areas in the spot tests, meaning only when high numbers of phage particles were present in a spot could lytic activity be observed. 

**Figure 1:**
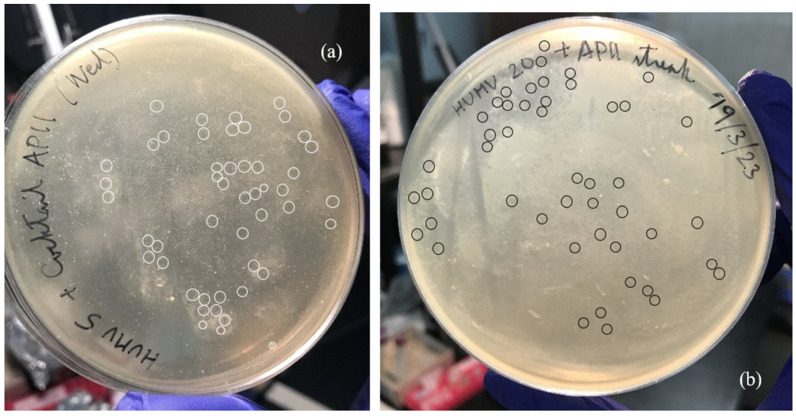
Activity assays of the AP11 cocktail on lawns of clinical isolates (**a**) HUMV 5 and (**b**) HUMV 20, with some of the most visible PFUs circled in white and black, respectively. Cocktail AP11 exhibits a greater tendency for temperateness towards HUMV 5 than towards HUMV 20, where PFUs appear less cloudy and more well-defined.

However, the clinical strains finally showed sensitivity to cocktail AP11. The Appelmans protocol so far has created a cocktail of phages that are lytic enough to produce PFUs against at least HUMV 5 and HUMV 20 (Group 1) (Figure 1), thus, with an EOP > 0, and a cocktail that is able to lyse HUMV 25, HUMV 29, and HUVH 7898 (Group 2) in liquid culture, which the parent cocktail was completely unable to. 

### 3.4. Genome Sequencing

Bioinformatic analysis revealed more than one genome in one of the phage libraries. The final phages that came out and their details are captured in Table 2 and Table 3.

## 4. Discussion

Of the 113 environmental phages isolated from *M. smegmatis* mc^2^ 155, only 16 showed any level of bactericidal activity against MABC strains. This low proportion is in tune with that around the globe [8]. All the environmental phages were highly lytic against *M. smegmatis* mc^2^ 155 but either non-infective or far too temperate against our MABC strains to present any PFUs. This trend is also reflected around the world, where MABC clinical isolates have varying phage susceptibilities, but there is hardly ever a phage naturally lytic towards MABC [34,35]. Phage Muddy demonstrates efficient infection and killing of certain MABC clinical isolates. But most phages that infect MABC, such as ZoeJ, BPs, Itos, D29, Faith1, Fionnbarth, Elmo, and TM4, are temperate, being efficient at infecting certain MABC strains at most, and poor killers of any of them [13,35,36]. 

Phages like Isca that are isolated on MABC as host and do not propagate on non-pathogenic hosts such as *M. smegmatis* [10] cannot be clinically applied as such due to regulatory obstacles [37]. Also, phages isolated from *M. smegmatis* that are temperate against MABC may only show lytic areas in the spot test when amplified to high titres and may not be visible in the preceding stages of phage isolation, activity assay, and titration if screened on MABC lawns. It is for these two reasons that the gold standard is to screen all environmental phages based on their exclusive ability to propagate on *M. smegmatis* mc^2^ 155 before testing them on clinical strains. However, it may also be viable to isolate phages from MABC as hosts that do not propagate on non-pathogenic hosts, and later directedly evolve them to make them do so. Such is the case of spontaneously released prophages [35]. Such phages may be more or less lytic towards certain clinical strains, but they likely do not also infect *M. smegmatis* without undergoing a due course of directed evolutionary action. 

MABC morphotype has been found to be linked to the growth medium to a certain extent, with LB agar favouring the R morphotype and 7H10 favouring the S morphotype [23]. The effect should be reflected in how phage susceptibility changes whether MABC strains are grown in one medium or the other. Effectively, LB agar-cultured clinical strains were susceptible to a wider range of phages than 7H10-cultured clinical strains, supporting the previous observation that S morphotypes are categorically harder to kill than R morphotypes owing to the presence of GPLs, which likely mask the receptors through which phages gain entry into and infect the bacteria [35].

In the absence of highly lytic mycobacteriophages, we undertook directed evolution to generate more powerful phages. 

Numerous past researchers have performed directed evolution of viruses [9,15,16,38,39,40,41]. There has also been a study that evolved mycobacteriophages on environmental mycobacteria [42], but to our knowledge, there are no studies published on the evolution of mycobacteriophages with pathogenic mycobacteria such as MABC, perhaps because mycobacteria are some of the slowest growers of bacteria, considerably slowing down any possible research. 

Our Appelmans protocol has generated AP13 and AP11′ phages, which are far more lytic than any of the parental phages but still temperate at best. Due to time constraints, our research had to stop short, but results are highly promising, and we hope to finish all 30 rounds of training at a future occasion. Due to the same time constraints, we had to isolate the evolved phages on the faster-growing *M. smegmatis*. By the intrinsic nature of the Appelmans protocol, in which phages only expand in host range and never lose their original host [15], we know that any evolved progeny that is isolable on an MABC host is also isolable on its original *M. smegmatis* host. In addition, by the time of AP13 and AP11′, the evolved phages far outnumber any parental phages that have not propagated on any clinical strains. Thus, the likelihood of isolating phages on an *M. smegmatis* lawn that cannot also form PFUs on MABC is intrinsically low. Nevertheless, we have not had the time to experimentally confirm that the isolated phages are also PFU-forming on MABC strains and may only claim so with a high degree of confidence. 

For sequencing, we randomly picked one parental phage sample and one evolved phage sample. We know that all mycobacteriophages are dsDNA [8]. Due to limited resources, our phage DNA was extracted using a Nextera tagmentation kit, which is not ideal, as it nicks the ends of genomes with sticky overhangs, leading to a loss of sequence data on the ends. The foolproof way to salvage this is with additional wet-lab work. Targeted Sanger sequencing can definitively resolve doubtful regions in the consensus sequence. Primer design, for that matter, is simple, as Consed readily creates a list of acceptable primers. However, it may be more cost-effective to redo genome sequencing with the appropriate DNA extraction kit [43]. A less robust, though potentially convincing, way to salvage this loss of data is by inference and deduction. Even the regions of lost data in the consensus sequence, though they experience a sudden drop in coverage, are always or almost always covered at the very least by sequence data from spontaneous ligation or concatemers, providing a minimum level of assurance of the actual data in those regions. If these data further match the known sticky overhangs of a sufficiently close neighbour in the same cluster, direct observation of end sequence may no longer be necessary. This is exactly our case, and we thus hereby specify that the sticky overhang sequences were inferred rather than experimentally determined. 

To our surprise, some of our phage libraries contained more than one genome, which indicates human error during phage isolation. Bioinformatic analyses also revealed the presence of many small contaminant contigs of *M. smegmatis* in all the sequenced samples, which implies the phage libraries were highly contaminated. These contaminants are likely the result of lysed bacterial nucleic acid debris that cannot be centrifuged or filtered out. Using DNases to cleanse the library of the non-encapsidated DNA in the first place could be a solution. Of course, the presence of contaminant contigs poses absolutely no obstacle to the bioinformatic post-processing of phage genomes, as Newbler is precisely designed to separate contaminant reads from actual phage reads by aligning them into separate contigs, which can then be easily visualised [43]. 

Mycobacteriophage gene functions are classified into three ranks. Rank 1 functions are obligatorily present in all genomes. Generically, they are terminase, portal protein, major capsid protein, and lysin A. Rank 2 functions are present in most genomes. Generically, they are HNH endonuclease, capsid maturation protease, scaffolding protein, and holin. Rank 3 functions are those that may or may not be identifiable. Generically, they are minor capsid protein, head-to-tail adaptor, head-to-tail-stopper, tail terminator, lysin B, tail fibre, and tail spike. In the *Siphoviridae* family, Rank 1 functions additionally include major tail protein, tape measure protein, and minor tail proteins, and Rank 3 functions additionally include tail assembly chaperones. In the *Myoviridae* family, Rank 1 functions additionally include tail tube protein and tail sheath protein, and Rank 3 functions additionally include baseplate protein/wedge/component. In the *Podoviridae* family, Rank 1 functions additionally include tail tube protein, and Rank 2 functions additionally include minor tail proteins (https://seaphagesbioinformatics.helpdocsonline.com/article-91, accessed on 31 December 2023). 

The parental phage Benzema belongs to subcluster D1, being highly similar to phage Nova. In subcluster D1, there are, as of now, 20 members, with an average genome size of 64,676 bp, an average GC content of 59.7%, an average of 86.9 genes, and no tRNAs. D1 genomes are circularly permuted and belong to the *Siphoviridae* family. These characteristics are in line with Benzema’s. Phage Nova has 88 genes, 27 (31%) of which have functional assignations. Amongst these, feature the capsid decoration protein, terminase, portal protein, JAB/MPN domain protein, minor capsid protein, major capsid protein, major tail protein, tail assembly chaperones, tape measure protein, minor tail protein, lysin A, lysin B, helicase, DNA binding protein, CDC45-like protein, oxidoreductase, deoxyribonucleotidase, methyltransferase, FabG-like protein, DNA polymerase III α subunit, ThyX-like thymidylate synthase, HNH endonuclease, and primase/polymerase [8]. Benzema has functional assignments to 41 of 86 genes (48%). They include capsid decoration protein, terminase, portal protein, capsid maturation protease, minor capsid proteins, scaffolding protein, major capsid protein, tail terminator, major tail protein, tail assembly chaperones, tape measure protein, minor tail proteins, lysin A, membrane proteins, DNA helicase, exonuclease, RecA-like DNA recombinase, oxidoreductase, 5′ nucleotidase, methyltransferase, FabG-like reductase, DnaE-like DNA polymerase III (α), ThyX-like thymidylate synthase, RuvC-like resolvase, helix-turn-helix DNA binding domain, HNH endonuclease, and DNA primase/polymerase. 

The progeny phage Suarez belongs to cluster O. In cluster O, there are, as of now, 28 members, with an average genome size of 71,124 bp, an average GC content of 65.4%, an average of 121 genes, and no tRNAs. Cluster O genomes have 3′ sticky overhangs and belong to the *Siphoviridae* family. These characteristics are in line with Suarez’s. Suarez is closely related to phages Dylan, Catdawg, Firecracker, and YungJamal, being closest to Dylan. Dylan has 121 genes, 31 (26%) of which have functional assignations, featuring DNA methylases, primase/polymerase, WhiB family transcription factor, terminases, portal protein, O-methyltransferase, glycosyltransferase, capsid maturation protease, capsid protein, head-to-tail stopper, major tail subunit, DNA binding protein, tail assembly chaperones, minor tail proteins, lysin A, lysin B, holin, helix-turn-helix DNA binding domain, DNA polymerase III sliding clamp (β), Ku-like dsDNA break-binding protein, ParB-like nuclease domain, and AAA-ATPase [8]. Suarez has functional calls to 45 (37%) of 122 genes, featuring DNA binding protein, DNA methyltransferases, endonuclease VII, RecB-like exonuclease/helicase, DNA polymerase/primase, WhiB family transcription factor, terminase large and small subunits, portal protein, O-methyltransferase, glycosyltransferases, galactosyltransferase, capsid maturation protease, major and minor capsid proteins, head-to-tail adaptor, major and minor tail proteins, helix-turn-helix DNA binding domains, tail assembly chaperones, tape measure protein, lysin A, lysin B, holin, membrane proteins, DNA polymerase III sliding clamp (β), Ku-like dsDNA break-binding protein, ParB-like nuclease domain, and AAA-ATPase. Cluster O phages have certain unique aspects, namely the singular prolate capsid, a non-canonical mechanism for the translation of the tape measure protein in Corndog, eight or nine putative SigA promoters, and a peculiar 17-bp motif of unknown function repeated over 30 times throughout the genome. The singular translation mechanism of Corndog’s tape measure protein, brought about by a ~600 bp non-coding gap prior to the *tmp* start site, is not shared by Suarez, which, much like Dylan, is able to translate tmp immediately downstream of the tail assembly chaperone genes. In contrast, the 17-bp motif, consisting of the palindromic repeat 5′-TGTTCGGNNNCCGAACA-3′, where N is either A or T, is also seen reiterated across Suarez [44].

The progeny phage PRodriguez belongs to subcluster B5 and is most similar to phage Donny. In subcluster B5, there are, as of now, 13 members, with an average genome size of 70,025 bp, an average GC content of 68.2%, an average of 96 genes, and no tRNAs. Subcluster B5 genomes are circularly permuted and belong to the *Siphoviridae* family. These properties are in consonance with PRodriguez. Donny has 97 genes, 27 (28%) having functional annotations, including ParB-like nuclease domain, glucosaminyl deacetylase, adenylate kinase, terminase, RuvC-like resolvase, portal protein, capsid maturation protease, major capsid hexamer and pentamer proteins, major and minor tail proteins, head-to-tail adaptor, tail assembly chaperone, tape measure protein, DNA binding proteins, lysin A, lysin B, DNA helicase, RepA-like helicase, DNA polymerase I, and HNH endonuclease [8]. PRodriguez has 32 (33%) of 97 genes functionally annotated, with features including ParB-like dsDNA partitioning protein, dpdA-like tRNA-guanine transglycosylase, galactosyltransferase, glucosaminyl deacetylase, adenylate kinase, terminase, RuvC-like resolvase, portal protein, capsid maturation protease, major capsid hexamer and pentamer proteins, membrane proteins, major tail protein, head-to-tail adaptor, tail assembly chaperones, tape measure protein, minor tail proteins, helix-turn-helix DNA binding domain, lysin A, serine hydrolase, exonuclease, DNA helicase, RepA-like helicase, DNA polymerase I, ribbon-helix-helix DNA binding domain, and HNH endonuclease. 

The progeny phage DiMaria belongs to subcluster A1 and is most similar to phage Tote. In subcluster A1, there are, as of now, 199 members, with an average genome size of 51,883 bp, an average GC content of 63.7%, an average of 91.1 genes, and no tRNAs. Subcluster A1 genomes have 3′ sticky overhangs and belong to the *Siphoviridae* family. These properties are in consonance with DiMaria. Tote has 37 (43%) of 87 genes with functions assigned, including HNH endonuclease, terminase (large and small subunits), minor tail proteins, lysin A and B, portal protein, capsid maturation protease, scaffolding protein, major capsid protein, head-to-tail adaptor, head-to-tail stopper, tail terminator, major tail protein, tail assembly chaperones, tape measure protein, minor tail proteins, serine integrase, DNA polymerase I, helix-turn-helix DNA binding domains, metallophosphoesterase, DNA primase, DNA methyltransferases, endonuclease VII, NrdH-like glutaredoxin, DnaB-like dsDNA helicase, Cas4 exonuclease, and immunity repressor [8]. DiMaria has 41 (46%) of 89 genes with assigned functions, including HNH endonucleases, terminase (large and small subunits), major tail proteins, lysin A and B, portal protein, capsid maturation protease, scaffolding protein, major capsid protein, head-to-tail adaptors, head-to-tail stopper, tail terminator, tail assembly chaperones, tape measure protein, minor tail proteins, serine integrase, DNA polymerase I, helix-turn-helix DNA binding domains, metallophosphoesterase, DNA primase, endonuclease VII, NrdH-like glutaredoxin, DnaB-like dsDNA helicase, DNA binding protein, RecB-like exonuclease/helicase, Imm-like superinfection immunity protein, immunity repressor, and DNA methyltransferase. Tote and DiMaria are both of temperate nature, at least on *M. smegmatis* lawns, as is evident from the presence of integrases. Cluster A1 is one of the 20 subclusters of cluster A, the largest cluster of actinobacteriophages [8]. Phage D29, famous for being used to develop shuttle phasmids, is an example of a subcluster A2 phage [45,46]. 

The variety of clusters presented by the four randomly sequenced phages and the even richer medley of genes found in each of the genomes lend support to the finding that phages constitute a continuum of genetic diversity in the constant exchange of genetic information [34,47]. Sequencing the rest of the parental phages would provide sufficient data to track from which parental phages the progeny phages inherited their genes. One thing is certain: the more the phages are trained, the more lytic they become. The overwhelming majority of genes, at the same time, are dark matter to our current knowledge, encoding for hypothetical proteins whose function only further experimental investigation will help disclose, particularly with the use of mass spectrometry [44], which will be essential to unlocking what exact molecular mechanisms make them increasingly aggressive and may clear the path to more efficient phage therapy. 

## 5. Conclusions

MABC clinical strains are rarely killed efficiently by environmental phages. Smooth strains are even harder to kill than rough strains. Incidentally, never before has the Appelmans protocol of directed evolution been successfully applied to mycobacteriophages against pathogenic mycobacteria, and our present work stands as the first unequivocal testament to the efficacy of this method in selecting, optimising, and fine-tuning mycobacteriophages for clinical therapy. The complete phage genome sequences will be fundamental for future molecular characterisations and for unveiling the mechanisms responsible for the sharpened lytic activity of evolved phages.

## Figures and Tables

**Table 1:** Breakdown of the bacterial strain subjects countered in the Appelmans protocol.

	Clinical Strains	Subspecies
Group 1	HUMV 5	*M. abscessus* subsp. *abscessus*
	HUMV 20	*M. abscessus* subsp. *massiliense*
	HUVH 8862	*M. abscessus* subsp. *bolletii*
Group 2	HUMV 25	*M. abscessus* subsp. *abscessus*
	HUMV 29	*M. abscessus* subsp. *abscessus*
	HUVH 7898	*M. abscessus* subsp. *massiliense*

**Table 2:** Properties of the four phages sequenced.

Library	Phage Name	Size (bp)	Avg. Coverage	Cluster	No. of Genes	%GC	Topology
25/9	Benzema	64,522	60×	D1	86	59.7	Circular
Φ13.3	Suarez	70,786	149×	O	122	65.5	Linear
	PRodriguez	70,064	92×	B5	97	68.4	Circular
	DiMaria	51,403	106×	A1	89	63.7	Linear

**Table 3:** The SRA, BioSample, BioProject, and GenBank accession numbers of the four phages sequenced.

Library	Phage Name	SRA Accession No.	BioSample Accession No.	BioProject Accession No.	GenBank Accession No.
25/9	Benzema	SRR27474740	SAMN39267258	PRJNA1061368	OR981606
Φ13.3	Suarez	SRR27474739	SAMN39267259		PP035217
	PRodriguez				PP035218
	DiMaria				PP062882

## Data Availability

Genomic data for the sequenced bacteriophages has been deposited in NCBI using the accession numbers provided in Table 3.

## Supplementary Materials

The following supporting information can be downloaded at https://www.mdpi.com/article/10.3390/microorganisms12020374/s1. Table S1. Phages isolated from Valencia’s sewage waters and their titres; Table S2. Phages isolated from Santander’s sewage waters and HUMV wastewaters, and their titres. Table S3. Spot test results of HUVH strains challenged with phages F6, F23, and F27 on 7H10 and with cocktail AP11 on LB agar. Table S4. HUMV strains challenged with phages F2, F6, F10, F15, F21, F23, and F27 on 7H10. Table S5. HUMV strains challenged with phages F2, F6, F10, F15, F21, F23, and F27, and with cocktail AP11 on LB agar. Table S6. Phages that were found active when assayed on representative subsp. abscessus, massiliense, and bolletii strains.
