# Supplementary material for: Complete Genome Sequences of Four Mycobacteriophages Involved in Directed Evolution against Undisputed Mycobacterium abscessus Clinical Strains
Source: Microorganisms. 2024 Feb 11;12(2):374. doi: 10.3390/microorganisms12020374 (PMC10893344; doi:10.3390/microorganisms12020374)
Supplement: Supplementary file 1 [file microorganisms-12-00374-s001.zip › microorganisms-2842320-supplementary.pdf]

Table S1. Phages isolated from Valencia's sewage waters and their titres.

Phage	Titre (PFU/ml)
F2	5.6E10
F6	1.12E11
F8A	5.5E7
F8B	6.5E9
F10	4E10
F15	5.5E10
F18	1.44E11
F21	2.84E10
F23	9.6E11
F27	2.24E11

Table S2. Phages isolated from Santander's sewage waters and HUMV wastewaters, and their titres.

Phage	Titre (PFU/ml)	Phage	Titre (PFU/ml)	Phage	Titre (PFU/ml)
JC 16/01	1.60E09	02/11	1.92E10	04/01	5.10E09
ARQ S1	4.00E05	03/11	4.00E08	08/01	2.76E10
HUMV Noche	6.00E03	06/11	1.30E09	09/01	7.40E09
08/09	4.00E08	07/11	5.10E09	10/01	4.00E08
09/09	1.70E09	08/11	1.00E10	11/01	2.72E10
18/09	1.20E17	09/11	2.31E10	12/01	2.00E09
19/09	5.00E15	10/11	1.92E10	15/01	1.00E08
20/09 (1)	2.43E10	13/11	5.64E10	16/01	1.00E10
20/09 (2)	3.60E15	14/11	7.00E09	17/01	3.00E08
22/09	2.00E08	15/11	3.80E07	18/01	1.00E08
25/09	1.74E10	16/11	3.50E09	19/01	1.50E07
26/09 (1)	1.30E15	17/11	5.00E08	22/01	6.00E04
26/09 (2)	1.20E09	20/11	1.35E10	23/01	3.00E11
28/09 (1)	1.00E10	21/11	2.56E10	24/01	1.20E11
28/09 (2)	2.16E10	22/11	1.12E11	25/01	1.00E08
02/10	4.00E10	23/11	1.17E11	26/01	5.00E05
03/10	4.00E04	24/11	1.47E11	29/01	4.00E03
04/10	3.60E10	27/11	2.00E08	30/01	1.20E10
05/10	2.90E09	29/11	5.20E09	31/01	3.60E05
06/10	3.00E08	30/11	3.80E07	01/02	3.00E09
09/10	4.00E04	04/12	8.40E09	05/02	5.00E04
10/10 (1)	1.64E10	06/12	3.1E9	06/02	5.00E09
10/10 (2)	9.00E08	08/12	9.32E10	07/02	7.00E08
12/10	1.46E10	11/12	8.71E10	08/02	1.00E09
16/10	7.00E04	12/12	1.70E09	09/02	2.50E09
17/10	1.00E08	13/12	1.90E09	12/02	5.00E09
18/10	3.00E08	14/12	7.08E10	13/02	4.00E09
19/10	6.00E08	15/12	3.10E09	14/02	4.60E09
20/10	1.01E08	18/12	6.00E06	15/02	4.20E09
23/10	3.00E04	21/12	2.30E09	16/02	3.00E09
24/10	3.20E09	22/12	1.40E07	19/02	3.00E05
25/10	2.80E09	29/12	7.00E08	20/02	2.80E05
26/10	8.80E10	01/01	2.80E09	21/02	4.50E09
27/10	1.12E11	02/01	3.52E06	22/02	2.00E05
01/11	1.57E10				

Table S3. Spot test results of HUVH strains challenged with phages F6, F23, and F27 on 7H10 and with cocktail AP11 on LB agar. 0, inactive; 1, active; blank, not tested.

HUVH Strain	Subspecies	F6	F23	F27	Cocktail AP11
6970	*M. abscessus* subsp. *bolletii*				1
7948	*M. abscessus* subsp. *abscessus*	1	1	1	
7898	*M. abscessus* subsp. *massiliense*				
8130	*M. abscessus* subsp. *abscessus*				1
8169	*M. abscessus* subsp. *abscessus*	1	1	1	
8153	*M. abscessus* subsp. *abscessus*				1
8261	*M. abscessus* subsp. *abscessus*	0	0	0	1
8449	*M. abscessus* subsp. *abscessus*	1	1	1	
8396	*M. abscessus* subsp. *abscessus*				1
8617	*M. abscessus* subsp. *abscessus*	1	1	1	
8874	*M. abscessus* subsp. *abscessus*				1
8727	*M. abscessus* subsp. *abscessus*				1
8862	*M. abscessus* subsp. *bolletii*	0	0	0	
8925	*M. abscessus* subsp. *abscessus*	1	1	1	
8982	*M. abscessus* subsp. *abscessus*	1	1	1	
9047	*M. abscessus* subsp. *abscessus*	1	1	1	
9080	*M. abscessus* subsp. *massiliense*	1	1	1	
9161	*M. abscessus* subsp. *abscessus*				1
9380	*M. abscessus* subsp. *abscessus*				1

Table S4. HUMV strains challenged with phages F2, F6, F10, F15, F21, F23, and F27 on 7H10. 0, inactive; 1, active; blank, not tested.

HUMV strain	Subspecies	F2	F6	F10	F15	F21	F23	F27
1	*M. abscessus* subsp. *abscessus*							
2	*M. abscessus* subsp. *abscessus*	1	1	1	0	0	1	1
3	*M. abscessus* subsp. *abscessus*	1	1	1	0	0	1	1
4	*M. abscessus* subsp. *abscessus*	1	1	1	0	0	1	1
5	*M. abscessus* subsp. *abscessus*	1	1	1	0	0	1	1
6	*M. abscessus* subsp. *abscessus*	0	1		0	0	1	1
7	*M. abscessus* subsp. *abscessus*	0	1		0	0	1	1
9	*M. abscessus* subsp. *abscessus*	0	1		0	0	1	1
10	*M. abscessus* subsp. *abscessus*	0	1		0	0	1	1
11	*M. abscessus* subsp. *abscessus*	0	1		0	0	1	1
12	*M. abscessus* subsp. *abscessus*	0	0		0	0	1	1
13	*M. abscessus* subsp. *abscessus*	0	1		0	0	1	1
14	*M. abscessus* subsp. *abscessus*	0	0		0	0	1	1
16	*M. abscessus* subsp. *abscessus*	0	1		0	0	1	1
18	*M. abscessus* subsp. *abscessus*	0	1		0	0	1	1
19	*M. abscessus* subsp. *abscessus*	0	1		0	0	1	1
20	*M. abscessus* subsp. *massiliense*	0	1		0	0	1	1
21	*M. abscessus* subsp. *abscessus*	0	1		0	0	1	1
22	*M. abscessus* subsp. *abscessus*	0	1		0	0	1	1
24	*M. abscessus* subsp. *abscessus*	0	1		0	0	1	1
25	*M. abscessus* subsp. *abscessus*	0	1		0	0	1	1
26	*M. abscessus* subsp. *abscessus*	0	1		0	0	1	1
27	*M. abscessus* subsp. *abscessus*	0	1		0	0	1	1

28	*M. abscessus* subsp. *abscessus*	1	1		0	0	1	1
29	*M. abscessus* subsp. *abscessus*	0	1		0	0	1	1

Table S5. HUMV strains challenged with phages F2, F6, F10, F15, F21, F23, and F27, and with cocktail AP11 on LB agar. 0, inactive; 1, active; blank, not tested.

HUMV strain	F2	F6	F10	F15	F21	F23	F27	Cocktail AP11
1								1
2	1	1	1	1	0	1	1	
3	1	1	1	0	0	1	1	
4	1	1	1	1	1	1	1	
5	1	1	1	1	0	1	1	
6	1	1		0	0	1	1	
7	1	1		0	0	1	1	
9	1	1		0	0	1	1	
10	1	1		0	0	1	1	
11	0	1		0	0	1	1	
12	1	1		0	0	1	1	
13	1	1		0	0	1	1	
14	0	1		0	0	1	1	
16	0	1		0	0	1	1	
18	0	1		0	0	1	1	
19	0	1		0	0	1	1	
20	0	1		0	0	1	1	
21	0	1		0	0	1	1	
22	0	1		0	0	1	1	
24	0	1		0	0	1	1	
25	0	1		0	0	1	1	
26	0	1		0	0	1	1	
27	0	1		0	0	1	1	
28	1	1		0	0	1	1	
29	0	1		0	0	1	1	

Table S6. Phages that were found active when assayed on representative subsp. *abscessus*, *massiliense*, and *bolletii* strains. Blank, unknown.

Phage	Clinical strain		
	**Subsp. *abscessus***	**Subsp. *massiliense***	**Subsp. *bolletii***
25/9	HUMV 5		HUVH 8862
26/9 (2)			HUVH 8862
12/10	HUMV 5	HUMV 20	HUVH 8862
24/10	HUMV 5	HUMV 20	HUVH 8862
14/11	HUMV 5		HUVH 8862
18/12	HUMV 5		HUVH 8862
21/12	HUMV 5, HUMV 25	HUMV 20	HUVH 8862
29/12	HUMV 5, HUMV 25	HUVH 7898	HUVH 8862

9/1	HUMV 5, HUMV 25		HUVH 8862
